# Accelerated knee osteoarthritis is associated with pre-radiographic degeneration of the extensor mechanism and cruciate ligaments: data from the Osteoarthritis Initiative

**DOI:** 10.1186/s12891-019-2685-y

**Published:** 2019-06-29

**Authors:** Julie E. Davis, Matthew S. Harkey, Robert J. Ward, James W. MacKay, Bing Lu, Lori Lyn Price, Charles B. Eaton, Grace H. Lo, Mary F. Barbe, Timothy E. McAlindon, Jeffrey B. Driban

**Affiliations:** 1 Division of Rheumatology, Allergy & Immunology, Tufts Medical Center, 800 Washington Street, Box #406, Boston, MA 02111 USA; 2 Department of Population and Quantitative Health Sciences, University of Massachusetts Medical School, 368 Plantation Street, Worcester, MA 01605 USA; 3 Department of Radiology, Tufts Medical Center, 800 Washington Street, Boston, MA 02111 USA; 4 Department of Radiology, University of Cambridge School of Clinical Medicine, Box 218, Level 5, Addenbrooke’s Hospital, Cambridge, CB2 0QQ UK; 5 Brigham & Women’s Hospital and Harvard Medical School, 75 Francis Street PBB-B3, Boston, MA 02115 USA; 6 The Institute for Clinical Research and Health Policy Studies, Tufts Medical Center, 800 Washington Street, Box #63, Boston, MA 02111 USA; 7 Tufts Clinical and Translational Science Institute, Tufts University, 800 Washington Street, Box #63, Boston, MA 02111 USA; 8 Center for Primary Care and Prevention, Alpert Medical School of Brown University, 111 Brewster Street, Pawtucket, RI 02860 USA; 9 Medical Care Line and Research Care Line, Houston Health Services Research and Development (HSR&D) Center of Excellence Michael E. DeBakey VAMC, Houston, TX USA; 10 Section of Immunology Allergy and Rheumatology, Baylor College of Medicine, Houston, TX. 1 Baylor Plaza, BCM-285, Houston, TX 77030 USA; 11 Department of Anatomy and Cell Biology, Temple University School of Medicine, 3500 North Broad Street, Philadelphia, PA 19140 USA

**Keywords:** Epidemiology, Knee, Osteoarthritis, Ligaments, Tendons

## Abstract

**Background:**

To determine if adults with incident accelerated knee osteoarthritis (KOA) are more likely to have degenerative knee ligaments or tendons compared to individuals with typical or no KOA.

**Methods:**

We identified 3 sex-matched groups among Osteoarthritis Initiative participants who had a knee without radiographic KOA at baseline (Kellgren-Lawrence [KL] < 2): 1) accelerated KOA: at least 1 knee had KL grade ≥ 3 in ≤48 months, 2) typical KOA: at least 1 knee increased in radiographic scoring within 48 months, 3) no KOA: both knees had the same KL grade at baseline and 48 months. We evaluated knee magnetic resonance images up to 2 years before and after a visit when the accelerated or typical KOA criteria were met (index visit). Radiologists reported degenerative signal changes for cruciate and collateral ligaments, and extensor mechanism and proximal gastrocnemius tendons. We used generalized linear mixed models with 2 independent variables: group and time.

**Results:**

Starting at least 2 years before onset, adults with accelerated KOA were twice as likely to have degenerative cruciate ligaments than no KOA (odds ratio = 2.10, 95% CI = 1.18, 3.74). A weaker association (not statistically significant) was detected for adults with accelerated versus typical KOA (OR = 1.72, 95%CI = 0.99, 3.02). Regardless of time, adults with accelerated (odds ratio = 2.13) or typical KOA (odds ratio = 2.16) were twice as likely to have a degenerative extensor mechanism than no KOA. No other structural features were statistically significant.

**Conclusions:**

Degenerative cruciate ligaments or extensor mechanism antedate radiographic onset of accelerated KOA. Hence, knee instability may precede accelerated KOA, which might help identify patients at high-risk for accelerated KOA and novel prevention strategies.

## Background

While knee osteoarthritis (KOA) is characteristically a slow progressing disorder, there is a subset of individuals that experience an abrupt and accelerated onset of radiographic advanced-stage disease, often less than 12 months [1–3]. Individuals that develop accelerated KOA report greater knee symptoms and functional limitations than those that develop the more common and gradual onset of KOA as early as 3 years before radiographic disease onset [2]. Additionally, those with accelerated KOA are 25 times more likely to receive a knee replacement over 9 years than those with a gradual onset of KOA [4]. Furthermore, the median time from the first evidence of radiographic progression to knee replacement is only 2.3 years, which highlights the short window for tertiary prevention [4]. It is critical that we understand the pathogenesis of accelerated KOA to develop strategies to identify high-risk patients, those with early-stage (pre-radiographic) accelerated KOA, and novel prevention strategies.

Degeneration of knee tendons and ligaments may antedate the onset of radiographic accelerated KOA and contribute to prodromal joint symptoms that are common to accelerated KOA. Degeneration of the cruciate ligaments are present in knees with KOA and may antedate KOA [5, 6]. Early degeneration of ligaments and tendons may be a sign of abnormal joint loading or instability, which increases shear force to the articular cartilage, may be an early sign of a person at risk for OA, and a risk factor for a knee injury that could precipitate accelerated KOA [7]. However, it remains unclear if the degenerative status of the ligaments and tendons in the knee are associated with the development of accelerated KOA.

We aimed to determine if incident accelerated KOA is preceded and characterized by the degeneration of cruciate or collateral ligaments, as well as extensor mechanism or proximal gastrocnemius tendons compared to those with no KOA or a gradual onset of KOA (typical KOA). The Osteoarthritis Initiative (OAI) offers a unique opportunity to study the relationship between degeneration of ligaments and tendons and the natural history of incident KOA, including accelerated KOA. If people with accelerated KOA exhibit unique degenerative ligament or tendon characteristics leading to the onset of disease, it may indicate accelerated KOA is preceded by maladaptive responses to overloading or instability. If so, this may help identify high-risk patients and novel prevention strategies.

## MethodS

We conducted a longitudinal analysis of individuals in the Osteoarthritis Initiative (OAI) using radiographs, magnetic resonance (MR) images, and clinical data at baseline and the first 4 annual follow-up visits. The OAI is a multicenter cohort study of 4796 adults with or at risk for symptomatic KOA. Staff at four clinical sites (Memorial Hospital of Rhode Island, University of Maryland and Johns Hopkins University, the University of Pittsburgh, and The Ohio State University) recruited participants between 2004 and 2006. OAI data (including images) and protocols are publicly available [8]. The OAI has met all criteria for ethical standards regarding human studies as described in the 1964 Declaration of Helsinki and all amendments. The OAI study was reviewed and approved by institutional review boards at each OAI clinical site and the OAI coordinating center (Memorial Hospital of Rhode Island Institutional Review Board, University of Maryland Baltimore – Institutional Review Board, University of Pittsburgh Institutional Review Board, and The Ohio State University’s Biomedical Sciences Institutional Review Board; Committee on Human Research at University of California, San Francisco; Approval Number: 10–00532). All participants offered written informed consent before starting the study.

### Participant selection

We classified 3 groups based on radiographic criteria. Adults with incident accelerated KOA included anyone with at least 1 knee that had no radiographic KOA at baseline (Kellgren-Lawrence [KL] grade < 2) that developed advanced-stage KOA (KL grade 3 or 4) within 48 months (*n* = 125) [3, 9]. Individuals with typical KOA had no baseline radiographic KOA (KL < 2) in both knees and 1 or more knee increased in radiographic scoring within 48 months (*n* = 187; excluding those with accelerated KOA). Individuals with no KOA had no radiographic KOA in both knees at baseline and had no change in KL grade in either knee during 48-months (*n* = 1325).

Our overall goal of the accelerated KOA project was to characterize accelerated KOA using imaging and biochemical biomarkers as well as an array of clinical outcomes (e.g., patient-reported outcomes, functional assessments). Hence, to ensure the feasibility of completing all of the biomarker assessments we did 1:1:1 matching based on sex (*n* = 125 participants/group).

### Index knee

For adults with incident accelerated or typical KOA, the index knee was the first knee to experience accelerated or typical KOA, respectively. For an individual with no KOA, we defined the index knee to be the same side as their matched member of the accelerated KOA group.

### Definition of index visit

**Fig. 1:**
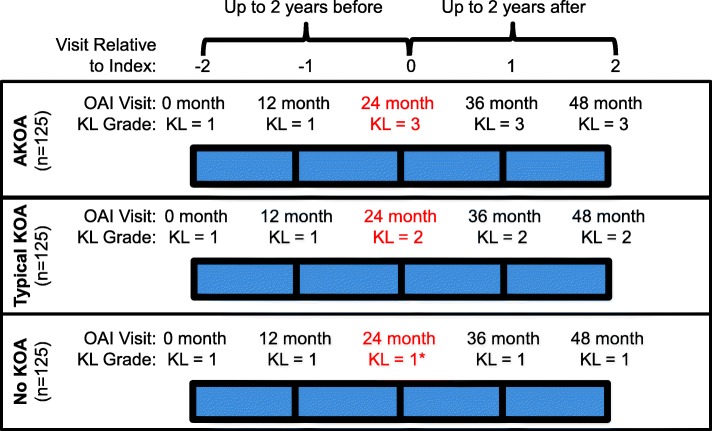
Examples of the Index Visit and Observation Period for a Member of Each Group. Key abbreviations: Accelerated Knee Osteoarthritis (AKOA), Knee Osteoarthritis (KOA), or No KOA. Kellgren-Lawrence (KL), Osteoarthritis Initiative (OAI). The red font represents the index visit (visit relative to index = 0). For individuals with accelerated or typical KOA, the index visit was the OAI visit when a person met the criteria for accelerated or typical KOA, respectively. * For someone with no KOA, the index visit was the same visit as that person’s matched member of the incident accelerated KOA group. The observation period was up to 2 years before and after the index visit (visit relative to index = −2 to 2 years)

For individuals with accelerated KOA, the index visit was the OAI visit when a participant met the criteria for accelerated KOA (i.e., visit when someone had KL = 3 or 4; potential OAI visits: 12-, 24-, 36-, or 48-month visit). For adults with typical KOA, the index visit was the OAI visit when a participant met the criteria for typical KOA (i.e., visit when someone first increased KL grade; potential OAI visits: 12-, 24-, 36-, or 48-month visit). For someone with no KOA, the index visit was the same OAI visit as that person’s matched member of the incident accelerated KOA group. Figure 1 illustrates examples of the index visit and observation period for a member of each group.

### Knee radiographs

To classify individuals into groups we relied on readings of bilateral weight-bearing, fixed-flexion posteroanterior knee radiographs. These images were obtained at baseline and annual follow-up visits. Blinded central readers recorded KL grades (0 to 4; weighted kappa = 0.70–0.80; files: kXR_SQ_BU##_SAS; file versions: 0.6, 1.6, 3.5, 5.5, and 6.3) [8].

### Magnetic resonance imaging

Magnetic resonance (MR) images were attained annually with one of four identical Siemens (Erlangen, Germany) Trio 3-Tesla MR systems at each OAI site. The musculoskeletal radiologists (RJW and JWM) performing semi-quantitative scoring were provided all the sequences acquired on each index knee at each visit; including a sagittal intermediate-weighted, turbo spin echo, fat-suppressed MR sequence with the following parameters: field of view = 160 mm, slice thickness = 3 mm, skip = 0 mm, flip angle = 180 degrees, echo time = 30 ms, recovery time = 3200 ms, 313 × 448 matrix, x resolution = 0.357 mm, y resolution = 0.511 mm, and total slice number = 37. The OAI sequences have been described elsewhere [8, 10].

### MR image readings

Two musculoskeletal radiologists reviewed MR images (RJW:255 cases, JWM:120 cases) to assess the integrity of the anterior and posterior cruciate ligaments, medial and lateral collateral ligaments, extensor mechanism (quadriceps femoris tendon and patellar ligament) [11], and gastrocnemius proximal tendons. Readers noted if the structures appeared normal or had degenerative pathologic findings. We defined a degenerative appearance as the existence of atypical intrinsic high-signal intensity in the substance of the ligaments or tendon without a discrete tear. Readers had good agreement among 25 cases: prevalence-adjusted and bias-adjusted kappa (PABAK) were 0.42 to 0.75: anterior cruciate ligament PABAK = 0.50, posterior cruciate ligament PABAK = 0.48, medial collateral ligament PABAK = 0.75, lateral collateral ligament PABAK = 0.67, extensor mechanism PABAK = 0.42, gastrocnemius proximal tendons PABAK = 0.42.

Since less than 10% of adults with no KOA (*n* < 6) had degeneration in the posterior cruciate ligament we analyzed the cruciate ligaments collectively as one outcome: cruciate ligament degeneration, which was defined as the presence of degenerative appearance in the anterior or posterior cruciate ligament. Similarly, less than 10% of adults with no KOA (*n* < 3) had degeneration in the lateral collateral ligament; hence, we analyzed the collateral ligaments collectively as one outcome: collateral ligament degeneration, which was defined as the presence of degenerative appearance in the medial or lateral collateral ligament.

### Statistical analysis

We ran descriptive statistics to assess baseline characteristics of the 3 groups. To determine any association between degeneration of the ligaments or tendons and group assignment, we used generalized linear mixed models assuming compound symmetry. Independent variables included group (3 levels), and time (up to 5 levels). We also assessed a group-by-time interaction. For our primary analyses we adjusted for sex (matching variable) [12] and factors associated with missing MR data at the next visit (i.e., age, body mass index, injury, frequent knee pain, days with limited activity in prior month, overall global rating, and WOMAC pain).

We conducted several sensitivity analyses: 1) adjusted only for sex, which was our matching factor, 2) among people who developed accelerated KOA and had no radiographic OA bilaterally at baseline (*n* = 54 per group with matched sample), 3) among individuals who developed accelerated KOA in < 12 months (*n* = 71 per group with matched sample), and 4) among those who developed typical KOA and had KL = 2, which is a common radiographic criteria for KOA (*n* = 76 per group with matched sample). We also performed one sensitivity analysis with no matching among people with complete data at every visit (accelerated KOA *n* = 12, typical KOA *n* = 29, no KOA *n* = 25). Since the sensitivity analyses were always among a smaller sample size, we performed them adjusted for sex only. All analyses were conducted with SAS Enterprise 7.15 (Cary, NC, USA).

## Results

### Descriptive characteristics

Table 1 offers an overview of the characteristics of the three groups. In brief, the groups were mostly female (63%), overweight, and 24 to 39% of participants in each group reported frequent knee pain during the prior 12 months.

**Table 1:** Descriptive Characteristics of those with Accelerated, Common, and No Knee Osteoarthritis (KOA) at Baseline

Variables (means, SD; except where noted)	AKOA (*n* = 125)	Typical KOA (*n* = 125)	No KOA (*n* = 125)
Females (n, %)	79 (63%)	79 (63%)	79 (63%)
Index knee KL Grade = 0 (n, %)	42 (34%)	71 (57%)	92 (74%)
Patellofemoral Osteoarthritis (MR-based)	88 (75%)	84 (69%)	80 (66%)
Frequent knee pain in past 12 months (n, %)	44 (35%)	49 (39%)	30 (24%)
Age (years)	62.5 (8.5)	58.4 (8.4)	57.3 (8.2)
Body mass index (kg/m^2^)	29.7 (4.6)	28.1 (4.4)	26.9 (4.4)
Global impact rating (0 to 10; higher score = greater impact)	1.7 (1.9)	1.1 (1.5)	0.8 (1.1)
How many days limited activities in past 30 days (0 to 30)?	3.2 (7.3)	1.7 (4.8)	1.4 (4.3)
WOMAC pain (0 to 20; higher score = more pain)	2.3 (3.1)	1.8 (2.3)	1.6 (2.4)

*AKOA* accelerated knee osteoarthritis, *KL* Kellgren-Lawrence, *MR* magnetic resonance, *WOMAC* Western Ontario and McMaster Universities Osteoarthritis Index

Table 2 highlights the frequency of the degenerative ligaments and tendons over time.

**Table 2:** Frequency of Degenerative Ligaments/Tendons Among Accelerated Knee Osteoarthritis (AKOA), Common Knee Osteoarthritis (KOA), No KOA

Frequency (n [%])	Visit	AKOA	Typical KOA	No KOA
Cruciate Ligaments	-2	39 (43)	14 (22)	25 (26)
(Anterior & Posterior)	−1	50 (43)	34 (28)	30 (24)
	Index	49 (47)	35 (28)	30 (24)
	1	41 (51)	33 (31)	25 (26)
	2	17 (47)	27 (33)	11 (21)
Anterior Cruciate Ligament^a^	−2	36 (40)	14 (22)	23 (24)
	−1	44 (38)	32 (26)	27 (22)
	Index	44 (42)	33 (27)	27 (22)
	1	35 (44)	31 (28)	22 (23)
	2	15 (42)	24 (29)	9 (17)
Posterior Cruciate Ligament^a^	−2	9 (10)	1 (2)	5 (5)
	−1	14 (12)	8 (6)	6 (5)
	Index	13 (12)	8 (6)	6 (5)
	1	12 (15)	7 (6)	6 (6)
	2	6 (17)	8 (10)	3 (6)
Collateral Ligaments	−2	22 (24)	6 (9)	12 (13)
(Medial & Lateral)	−1	28 (24)	19 (15)	17 (14)
	Index	28 (26)	19 (15)	17 (14)
	1	24 (30)	20 (18)	15 (15)
	2	8 (22)	17 (20)	8 (15)
Medial Collateral Ligament^a^	−2	19 (21)	5 (8)	11 (11)
	−1	24 (11)	16 (13)	16 (13)
	Index	24 (23)	16 (13)	16 (13)
	1	23 (29)	17 (15)	14 (14)
	2	8 (22)	14 (17)	8 (15)
Lateral Collateral Ligament^a^	−2	5 (5)	3 (5)	3 (3)
	−1	7 (6)	6 (5)	3 (2)
	Index	7 (7)	6 (5)	3 (2)
	1	4 (5)	6 (5)	2 (2)
	2	2 (6)	6 (7)	0 (0)
Extensor Mechanism	−2	40 (43)	26 (40)	21 (22)
	−1	50 (43)	48 (39)	30 (24)
	Index	47 (45)	51 (41)	32 (26)
	1	36 (46)	46 (42)	26 (27)
	2	11 (31)	33 (39)	17 (32)
Proximal Gastrocnemius Tendon	−2	46 (50)	25 (38)	39 (41)
	−1	54 (46)	54 (44)	51 (41)
	Index	50 (47)	56 (45)	51 (41)
	1	37 (46)	50 (45)	41 (42)
	2	15 (42)	40 (48)	20 (38)

^a^The sum of the prevalence for each specific ligaments will not equal the overall cruciate or collateral ligament prevalence because some knees had anterior and posterior cruciate ligament degeneration or medial and lateral collateral ligament degeneration

We detected a significant group effect – but no group-by-time interaction – for the presence of degenerative cruciate ligaments (group *p* = 0.03; interaction *p* = 0.24) and degenerative extensor mechanism (group *p* = 0.01; interaction *p* = 0.43). We observed no significant associations with group nor group-by-time interaction with the presence of degenerative collateral ligaments (group *p* = 0.43; interaction *p* = 0.06) and proximal gastrocnemius tendon degeneration (group *p* = 0.71; interaction *p* = 0.12).

Starting at 2 years prior to the index visit, the presence of degenerative cruciate ligaments was stable over time: ranging from 43 to 51% among those with accelerated KOA, 22 to 33% among those with typical KOA, and 21 to 26% among people with no KOA. Regardless of time, adults with accelerated KOA were twice as likely to have degenerative cruciate ligaments than those with no KOA (odds ratio [OR] = 2.10, 95% confidence interval [CI] = 1.18 to 3.74). We found a weaker association, which was not statistically significant, for those with accelerated KOA versus those with typical KOA (OR = 1.73, 95% CI = 0.99 to 3.02). Adults with typical KOA had a similar odds of having a degenerative cruciate ligament as those with no KOA (OR = 1.21, 95% CI = 0.68 to 2.17). The sensitivity analyses supported an association between degenerative cruciate ligaments and accelerated KOA (accelerated KOA vs No KOA OR range: 2.59 to 6.28; accelerated KOA vs typical KOA OR range: 1.68 to 5.38) with the highest OR among participants with complete data. Similarly, the sensitivity analyses typically supported the finding that degenerative cruciate ligaments had a weak or no association with typical KOA (typical KOA vs No KOA OR range: 0.66 to 1.22) with the exception of a potential association among those who developed typical KOA with KL = 2 (OR = 1.85, 95% CI = 0.88 to 3.92).

Starting at 2 years prior to the index visit, the presence of degenerative extensor mechanism was stable over time: ranging from 31 to 43% among those with accelerated KOA, 39 to 42% among those with typical KOA, and 22 to 32% among people with no KOA. Regardless of time, adults with accelerated or typical KOA were twice as likely to have degenerative extensor mechanism than those with no KOA (accelerated KOA vs no KOA OR = 2.13, 95% CI = 1.19 to 3.82; typical KOA vs no KOA OR = 2.16, 95% CI = 1.23 to 3.79). Neither people with accelerated nor typical KOA were more likely to have a degenerative extensor mechanism than their peers (accelerated vs typical KOA OR = 0.99, 95% CI = 0.57 to 1.70). The sensitivity analyses showed similar results (OR accelerated KOA vs No KOA OR range: 1.37 to 2.88; typical KOA vs No KOA OR range: 1.32 to 2.92; accelerated KOA vs typical KOA OR range: 0.99 to 1.50).

## Discussion

Adults with incident KOA (accelerated or typical KOA) were more likely to have a degenerative extensor mechanism than those with no KOA. Furthermore, adults with accelerated KOA were more likely to present with degenerative cruciate ligaments than those with no KOA, regardless of time. The degenerative changes were evident at least 2 years prior to meeting the criteria for incident disease and remained stable over time. Hence, these changes may be early signs that can extend our understanding of the pathogenesis of KOA, especially accelerated KOA. These findings may offer novel insights to recognize adults at-risk for or with early-stage accelerated KOA and help identify prevention strategies.

The current findings supported previous observations that there is an association between degenerative cruciate ligaments and symptomatic KOA within the OAI [13]. However, our results expanded on those findings by demonstrating that cruciate ligament degeneration was more associated with incident accelerated KOA than a gradual onset of KOA. The cruciate ligament degeneration, which is characterized by altered fiber arrangement and collagen composition, may compromise ligamentous function and lead to instability and altered joint loading [5, 14]. In fact, a degenerative cruciate ligament may have similar consequences to a joint as a complete tear. For example, individuals with ACL degeneration and individuals with an ACL rupture presented with a greater severity of structural pathology (e.g., cartilage damage, bone marrow lesions) when compared to individuals with a normal ACL; however, there were no differences in these pathologies between adults with degenerative or acute ACL pathology [7]. These structural changes may be attributable to rotational and antero-posterior instability and increased external adduction moments that occur during walking [15, 16]. These biomechanical changes may increase shear forces and medial compartment loading, which could contribute to accelerated KOA [15, 16]. The degenerative cruciate ligaments among those with accelerated KOA may indicate that this rotational or anteroposterior instability stresses the knee prior to the onset of disease. Additionally, cruciate ligament degeneration may also place an individual at greater risk for a knee injury, which may compound the risk of developing accelerated KOA [13]. Adults with accelerated KOA may be distinct from those with typical KOA and warrant greater consideration in both clinical and research settings [2, 17–20]. Early detection of cruciate ligament degeneration may help clinicians and researchers identify people at risk for or with early-stage accelerated KOA and enable them to implement biomechanical interventions (e.g., gait retraining, bracing) or exercise programs that reduce the risk of injury or falls (e.g. neuromuscular training).

The role of a degenerative extensor mechanism in KOA has been less studied and the significance of it among those with accelerated or typical KOA should be further explored. While multiple structures can help distribute the load during knee flexion (e.g., proximal gastrocnemius tendons), the extensor mechanism is the sole mechanism for knee extension. Therefore, more direct loading to the extensor may contribute to the development of KOA; however, the cause and clinical implications of extensor mechanism degeneration (e.g., implications for strength testing and therapeutic exercises) warrants further study.

While this study characterized which ligamentous and tendinous structures antedated the onset of accelerated KOA, we acknowledge important limitations. For example, our inter-reader agreement for detecting the presence of degenerative signal changes in the ligaments and tendons ranged from moderate to good. If we had a stronger level of inter-reader agreement, we may have observed stronger associations between these pathologic findings and accelerated or typical KOA. Despite our inter-reader agreement, we observed significant associations between degenerative extensor mechanisms and cruciate ligaments with accelerated KOA. We also had missing MR data that could effect the results, particularly 2 years after the index visit. However, we performed robust analyses that adjusted for variables related to missing MR data. We also performed several sensitivity analyses to explore the robustness of these findings. Our study was also limited by the number of adults in each group. Despite this, the OAI offered an extraordinary chance to study the natural history of accelerated KOA.

## Conclusions

Starting as early as 2 years prior to radiographic onset of disease, adults with incident KOA were twice as likely to have a degenerative extensor mechanism. Furthermore, adults with accelerated KOA were more likely to have degenerative cruciate ligaments, which could be the result of excessive overloading or contribute altered joint mechanics that may increase the chance of accelerated KOA. Identifying people with these degenerative structures using MR imaging prior to the onset of KOA may help us target prevention strategies to help ameliorate the risk of KOA, especially accelerated KOA.

## Data Availability

The datasets analyzed during current study are available in the OAI repository, https://nda.nih.gov/oai/. The MR imaging readings will be made publicly available via the OAI repository or by contacting the corresponding author.

## Abbreviations

ACL: Anterior cruciate ligament
AKOA: Accelerated knee osteoarthritis
CI: Confidence interval
KL: Kellgren-Lawrence
KOA: Knee osteoarthritis
MR: Magnetic resonance
OAI: Osteoarthritis Initiative
OR: Odds ratio
PABAK: Prevalence-adjusted and bias-adjusted kappa
PCL: Posterior cruciate ligament
